# Supplementary material for: Student and teacher performance during COVID-19 lockdown: An investigation of associated features and complex interactions using multiple data sources
Source: PLoS One. 2023 Oct 25;18(10):e0291689. doi: 10.1371/journal.pone.0291689 (PMC10599549; doi:10.1371/journal.pone.0291689)
Supplement: S1 Fig — (PDF) [file pone.0291689.s001.pdf]

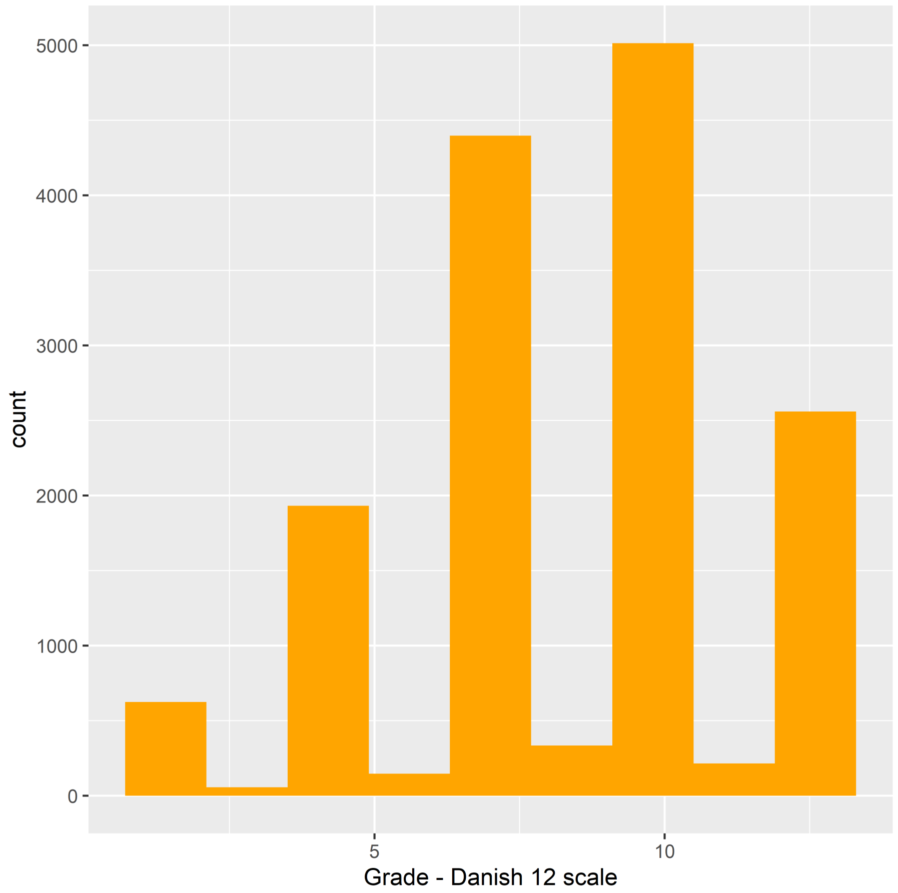

S1 Fig. Histogram of the grade response variable. The grade is based on an average of all graded activities within a course, and therefore some students at some courses receives grades between the normal grades, 2,4,7, 10 and 12
