# Supplementary figures and images for: Student and teacher performance during COVID-19 lockdown: An investigation of associated features and complex interactions using multiple data sources
Source: PLoS One. 2023 Oct 25;18(10):e0291689. doi: 10.1371/journal.pone.0291689 (PMC10599549; doi:10.1371/journal.pone.0291689)

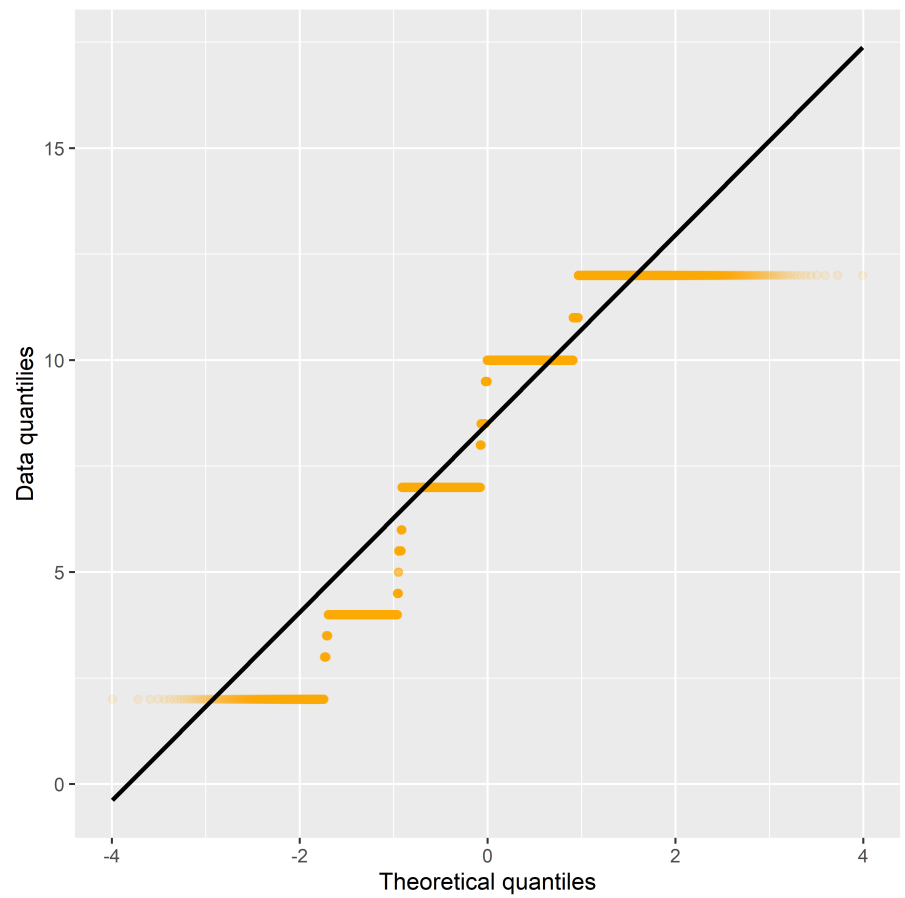

**S2 Fig.** QQ-plot of the grade target variable.

Supplement: S2 Fig — (PDF) [file pone.0291689.s002.pdf]

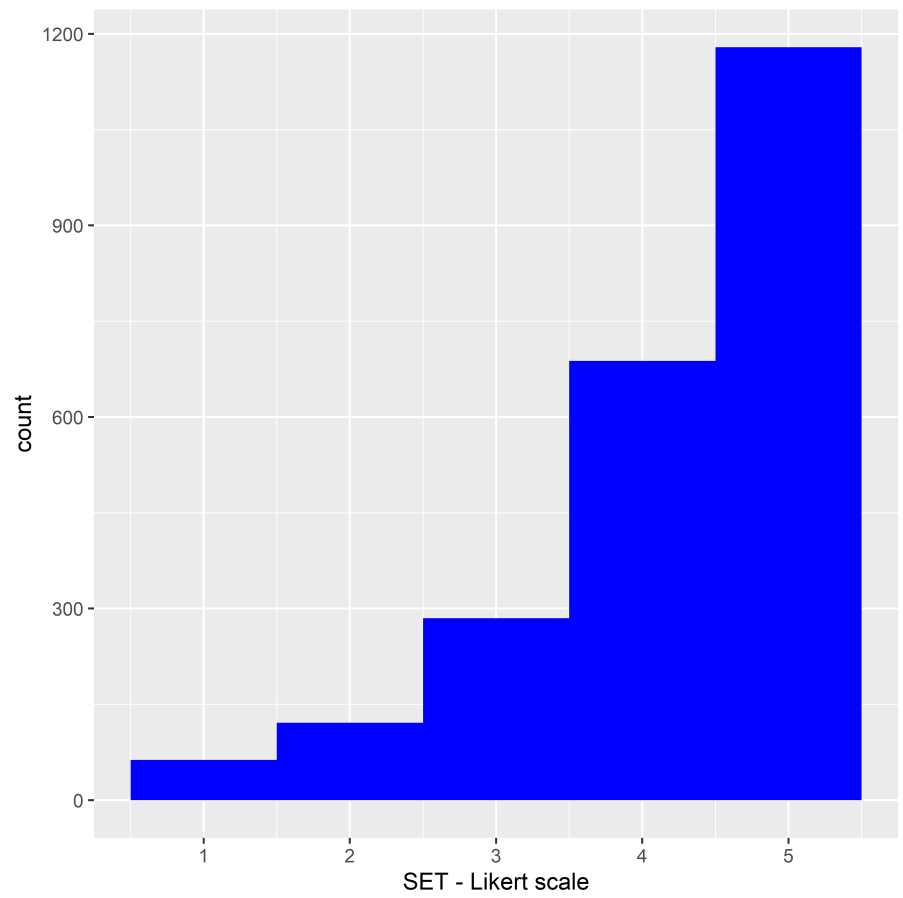

**S3 Fig. Histogram of the SET target variable.**

Supplement: S3 Fig — (PDF) [file pone.0291689.s003.pdf]

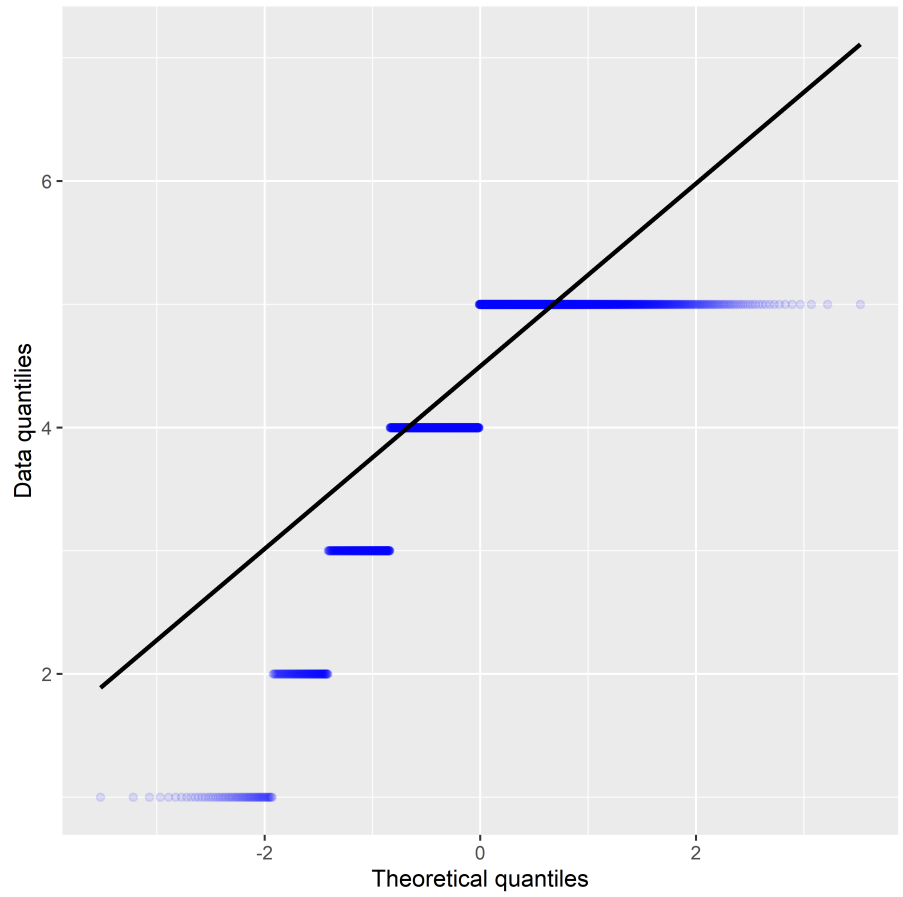

S4 Fig. QQ-plot of the SET response variable.

Supplement: S4 Fig — (PDF) [file pone.0291689.s004.pdf]
