# Supplementary material for: Student and teacher performance during COVID-19 lockdown: An investigation of associated features and complex interactions using multiple data sources
Source: PLoS One. 2023 Oct 25;18(10):e0291689. doi: 10.1371/journal.pone.0291689 (PMC10599549; doi:10.1371/journal.pone.0291689)
Supplement: S1 Table — (PDF) [file pone.0291689.s005.pdf]

S1 Table. Selected questions for the educators' survey. In italic are the feature names used in this publication.

Level	Code	Question	Answer Type
Course	Q25	To what extent had you already included online activities in this course prior to the lockdown? *Course had online elements, teacher*	Likert
	Q27	Has the switch to online teaching had an effect on the consistency between the course content and the subject's academic learning objectives? The consistency after the switch is... *Course stayed consistent, teacher*	Likert
	Q29	To what extent have you managed to use your original teaching design? *Course kept teaching design, teacher*	Likert
	Q30	How did the switch to online teaching affect the course in general compared to normal? The course became... *Course affected in general, teacher*	Likert
	Q31	How would you rate student performance compared to normal? *Assessment of students in course, teacher*	Likert
	Q32	Have you spent more or less time on teaching this course than usual/expected? *Time use on course, teacher*	Likert
	Q37	Is the actual exam's format meaningful in terms of the curriculum? *Course exam format meaningful, teacher*	Likert
Personal	Q3_1	Do you have a home workstation that provides a reasonable working position? *Physical working conditions, teacher*	Likert
	Q3_2	Do you have a home workstation that allows you to work in peace and quiet (e.g. by closing the door to another room)? *Quiet working conditions, teacher*	Likert
	Q6	(If children) How much of the share of time spent caring for the children and home schooling has been yours? *Share of time spent caring for children, teacher*	Likert
	Q7	Have you all in all been able to work as many hours as you usually do? *Time spend on work, teacher*	Likert
	Q8	Have you been able to work as efficiently as you usually do? *Self-assessed efficiency, teacher*	Likert
	Q9	Have you had family or other close relationships that you worry about, or have you been worried about getting sick yourself? *COVID-19 anxiety, teacher*	Likert
	Q14	What types of online tools did you have experience with in teaching prior to the COVID-19 lockdown? (More than one can be chosen) *Experience with (...), teacher*	Categorical dummies
	Q15	How would you rate your own IT technical skills (i.e. the application of IT used in online teaching)? *Technical skills, teacher*	Likert
	Q17	What was your attitude towards online/blended learning prior to the COVID-19 lockdown? *Attitude toward online teaching, teacher*	Likert
	Q169	Have you consulted with co-workers about your or their online activities? *Peer consulting, teacher*	Categorical into dummy
	Q172	Did you contact a support unit for help to switch your teaching? If so, how would you rate the received support? *Support from(...), teacher*	Likert
	Q175	What do you plan to use next semester, while there are still a requirement to teach 50% online? *Will use (...), teacher*	Categorical dummies
Comments		Comments received on exam change, teaching, advise to colleagues, personal factors as well as 14 in depth interviews.	
