# Supplementary material for: Student and teacher performance during COVID-19 lockdown: An investigation of associated features and complex interactions using multiple data sources
Source: PLoS One. 2023 Oct 25;18(10):e0291689. doi: 10.1371/journal.pone.0291689 (PMC10599549; doi:10.1371/journal.pone.0291689)
Supplement: S2 Table — (PDF) [file pone.0291689.s006.pdf]

S2 Table. Demographics of teachers in the grade model.

Staff type	N	Share who answered	Mean age	Mean #courses	Female share	International share
Part time	51	11%	51.9 (45.8)	1.5 (1.4)	27% (28%)	20% (15%)
Junior fac.	18	24%	34.0 (34.4)	1.5 (1.4)	67% (59%)	61% (55%)
Assist. Prof.	17	28%	36.1 (37.5)	1.8 (1.9)	29% (42%)	82% (68%)
Assoc. Prof.	55	37%	47.8 (46.9)	2.3 (2.1)	31% (30%)	33% (44%)
Professor	21	21%	56.4 (53.0)	2.3 (2.2)	10% (17%)	14% (39%)

Teachers in the grade model data were slightly older and taught slightly more courses compared to the background population ($p < 0.05$).
