# Supplementary material for: Student and teacher performance during COVID-19 lockdown: An investigation of associated features and complex interactions using multiple data sources
Source: PLoS One. 2023 Oct 25;18(10):e0291689. doi: 10.1371/journal.pone.0291689 (PMC10599549; doi:10.1371/journal.pone.0291689)
Supplement: S3 Table — (PDF) [file pone.0291689.s007.pdf]

S3 Table. Demographics for teachers in the SET-score model.

Staff type	N	Share who answered	Mean age	Mean #courses	Female share	International share
Part time	32	7%	52.7 (45.8)	1.6 (1.4)	25% (28%)	16% (15%)
Junior fac.	5	7%	35.6 (34.4)	1.6 (1.4)	40% (59%)	80% (55%)
Assist. Prof.	10	17%	33.6 (37.5)	2.0 (1.9)	40% (42%)	90% (68%)
Assoc. Prof.	46	31%	47.4 (46.9)	2.3 (2.1)	30% (30%)	33% (44%)
Professor	21	21%	56.4 (53.0)	2.3 (2.2)	10% (17%)	14% (39%)

Teachers in the model data were slightly older and taught slightly more courses compared to the background population ($p < 0.05$).
