# Supplementary material for: Student and teacher performance during COVID-19 lockdown: An investigation of associated features and complex interactions using multiple data sources
Source: PLoS One. 2023 Oct 25;18(10):e0291689. doi: 10.1371/journal.pone.0291689 (PMC10599549; doi:10.1371/journal.pone.0291689)
Supplement: S4 Table — (PDF) [file pone.0291689.s008.pdf]

S4 Table. Background demographics of the students included in the grade models.

Level of student	Year of admission	N	Share of pop.	Mean age	Female share	International share
Bachelor	2017	771	38%	23.7 (23.9)	54% (51%)	21% (15%)
Bachelor	2018	1542	67%	22.8 (23.0)	50% (46%)	12% (15%)
Bachelor	2019	1917	72%	21.8 (22.2)	43% (46%)	12% (14%)
Master	2018	288	9%	26.6 (26.3)	42% (51%)	33% (39%)
Master	2019	2095	69%	25.0 (25.4)	47% (50%)	35% (36%)

The few students started in earlier years are not displayed in the table and data for all enrolled students in brackets.
