# Supplementary material for: Student and teacher performance during COVID-19 lockdown: An investigation of associated features and complex interactions using multiple data sources
Source: PLoS One. 2023 Oct 25;18(10):e0291689. doi: 10.1371/journal.pone.0291689 (PMC10599549; doi:10.1371/journal.pone.0291689)
Supplement: S5 Table — (PDF) [file pone.0291689.s009.pdf]

S5 Table. Background demographics of the students included in the SET-score model.

Level of student	Year of admission	N	Share of pop.	Mean age	Female share	International share
Bachelor	2017	175	9%	23.7 (23.9)	66% (51%)	20% (15%)
Bachelor	2018	421	18%	22.6 (23.0)	55% (46%)	12% (15%)
Bachelor	2019	337	13%	21.7 (22.2)	55% (46%)	18% (14%)
Master	2018	36	1%	26.6 (26.3)	47% (51%)	36% (39%)
Master	2019	596	20%	24.8 (25.4)	58% (50%)	45% (36%)

The few students started in earlier years are not displayed in the table. Data for all students are in brackets.
