# Supplementary material for: Student and teacher performance during COVID-19 lockdown: An investigation of associated features and complex interactions using multiple data sources
Source: PLoS One. 2023 Oct 25;18(10):e0291689. doi: 10.1371/journal.pone.0291689 (PMC10599549; doi:10.1371/journal.pone.0291689)
Supplement: S6 Table — (PDF) [file pone.0291689.s010.pdf]

S6 Table. Table of linear regression coefficients, grade model.

term	estimate	std.error	p.value
Age, student	-0.32	0.05	0.0000
Age, teacher	-0.08	0.09	0.3685
Number of courses, teacher	0.05	0.07	0.5058
Bachelor level, student	0.07	0.08	0.4121
Children, teacher	0.70	0.09	0.0000
Danish citizen, teacher	-0.05	0.08	0.5698
Danish citizen, student	0.13	0.06	0.0250
Female, student	-0.03	0.04	0.4111
Female, teacher	0.21	0.07	0.0035
Course taught in Q4, teacher	-0.37	0.11	0.0008
Mandatory group work, student	0.18	0.08	0.0243
Previous year's GPA, course	1.37	0.06	0.0000
Exam from oral on campus to written at home, course	-0.87	0.08	0.0000
Historical GPA, student	2.07	0.04	0.0000
Assistant professor, teacher	0.30	0.07	0.0000
Part-time lecturer, teacher	0.14	0.08	0.0860
Professor, teacher	-0.41	0.07	0.0000
Postdoc/PhD, teacher	-0.03	0.06	0.6199
Support from department, teacher	0.63	0.08	0.0000
Support from IT unit, teacher	0.09	0.07	0.1875
Support from study board, teacher	-0.29	0.07	0.0001
Support from T&L unit, teacher	-0.19	0.07	0.0061
Peer consulting, teacher	0.59	0.10	0.0000
Course taught in Q3, teacher	0.01	0.06	0.8245
Experience with fully online courses, teacher	-0.29	0.12	0.0142
Experience with own videos, teacher	0.53	0.07	0.0000
Experience with others' videos, teacher	-0.55	0.08	0.0000
Experience with streamed lectures, teacher	0.12	0.07	0.1048
Experience with quizzes, teaches	0.29	0.08	0.0002
Experience with online forums, teacher	-0.16	0.07	0.0259
Experience with shared documents, teacher	0.37	0.07	0.0000
Experience with other tools, teacher	-0.09	0.09	0.2764
Technical skills, teacher	-0.35	0.07	0.0000
Attitude toward online teaching, teacher	-0.57	0.08	0.0000
Will use no tools in future, teacher	0.69	0.19	0.0003
Will use video recordings in future, teacher	0.32	0.07	0.0000
Will use streamed lectures in future, teacher	0.02	0.06	0.7000
Will use others' videos in future, teacher	0.02	0.07	0.7777
Will use quizzes in future, teacher	0.04	0.07	0.5546
Will use online forums in future, teacher	0.03	0.07	0.6864
Will use other tools in future, teacher	-0.37	0.07	0.0000
Course had online elements, teacher	0.00	0.06	0.9676
Course stayed consistent, teacher	0.10	0.07	0.1502
Course kept teaching design, teacher	0.38	0.06	0.0000
Physical working conditions, teacher	0.05	0.07	0.4540
Quiet working conditions, teacher	-0.42	0.07	0.0000
Course affected in general, teacher	-0.13	0.06	0.0350
Assessment of students in course, teacher	0.13	0.06	0.0248
Time use on course, teacher	0.14	0.05	0.0067
Course exam format meaningful, teacher	-0.29	0.06	0.0000
Share of time spent caring for children, teacher	-0.78	0.10	0.0000
Time spend on work, teacher	-0.09	0.07	0.1783
Self-assessed efficiency, teacher	-0.07	0.07	0.3467
COVID-19 anxiety, teacher	-0.14	0.06	0.0262
Exam from written on campus to at home, course	-0.36	0.08	0.0000
Enrollment year, student	0.25	0.07	0.0002
(Intercept)	-382.97	102.10	0.0002
