# Supplementary material for: Student and teacher performance during COVID-19 lockdown: An investigation of associated features and complex interactions using multiple data sources
Source: PLoS One. 2023 Oct 25;18(10):e0291689. doi: 10.1371/journal.pone.0291689 (PMC10599549; doi:10.1371/journal.pone.0291689)
Supplement: S7 Table — (PDF) [file pone.0291689.s011.pdf]

S7 Table. Table of linear regression coefficients, SET-score model.

term	estimate	std.error	p.value
Age, student	-0.03	0.04	0.4167
Age, teacher	0.20	0.08	0.0124
Number of courses taught, teacher	0.07	0.05	0.2116
Bachelor level, student	-0.22	0.07	0.0017
Children, teacher	0.01	0.08	0.8705
Danish citizen, teacher	-0.31	0.07	0.0000
Danish citizen, student	0.09	0.04	0.0254
Female, student	0.08	0.03	0.0108
Female, teacher	-0.10	0.07	0.1766
Course taught in Q4, teacher	-0.15	0.08	0.0603
Mandatory group work, student	-0.05	0.06	0.4536
Exam from oral on campus to written at home, course	-0.08	0.07	0.2652
Assistant professor, teacher	0.19	0.06	0.0022
Part-time lecturer, teacher	0.17	0.06	0.0037
Professor, teacher	0.05	0.06	0.4242
Postdoc/PhD, teacher	0.16	0.06	0.0042
Support from department, teacher	-0.01	0.07	0.8983
Support from IT unit, teacher	-0.15	0.07	0.0250
Support from study board, teacher	-0.06	0.06	0.3399
Support from T&L unit, teacher	0.10	0.06	0.1124
Peer consulting, teacher	-0.06	0.07	0.4191
Course taught in Q3, teacher	-0.13	0.05	0.0137
Experience with fully online courses	-0.02	0.10	0.8231
Experience with own videos, teacher	-0.02	0.06	0.7087
Experience with others' videos, teacher	0.10	0.06	0.0713
Experience with streamed lectures, teacher	-0.11	0.06	0.0715
Experience with quizzes, teacher	-0.10	0.07	0.1164
Experience with online forums, teacher	-0.03	0.06	0.6692
Experience with shared documents, teacher	-0.09	0.06	0.1296
Experience with other, teacher	-0.07	0.07	0.3220
Technical skills, teacher	0.03	0.07	0.6686
Attitude towards online teaching, teacher	0.22	0.07	0.0014
Will use no tools in future, teacher	-0.46	0.16	0.0043
Will use video recordings in future, teacher	0.13	0.06	0.0244
Will use streamed lectures in future, teacher	0.06	0.05	0.1960
Will use others' videos in future, teacher	-0.21	0.06	0.0003
Will use Quizzes in future, teacher	0.03	0.05	0.6087
Will use online forums in future, teacher	-0.15	0.05	0.0037
Will use other tools in future, teacher	0.06	0.06	0.3389
Course had online elements, teacher	0.12	0.05	0.0239
Course stayed consistent, teacher	0.01	0.06	0.9047
Course kept teaching design, teacher	0.03	0.06	0.5351
Physical work conditions, teacher	-0.01	0.05	0.8576
Quiet work conditions, teacher	-0.18	0.06	0.0014
Course affected in general, teacher	-0.09	0.06	0.0987
Assessment of students in course, teacher	0.19	0.05	0.0001
Time use on course, teacher	0.12	0.05	0.0217
Course exam format meaningful, teacher	-0.07	0.07	0.3085
Share of time spent caring for children, teacher	0.06	0.10	0.5143
Time spend on work, teacher	0.25	0.08	0.0010
Self-assessed efficiency, teacher	-0.07	0.06	0.2462
COVID-19 anxiety, teacher	0.08	0.05	0.1254
Exam from written on campus to at home, course	-0.07	0.07	0.2756
Enrollment year, student	-0.07	0.05	0.1930
SET score, course	1.20	0.03	0.0000
Historical SET score, teacher	0.42	0.05	0.0000
(Intercept)	106.19	81.89	0.1949
