# Supplementary material for: Student and teacher performance during COVID-19 lockdown: An investigation of associated features and complex interactions using multiple data sources
Source: PLoS One. 2023 Oct 25;18(10):e0291689. doi: 10.1371/journal.pone.0291689 (PMC10599549; doi:10.1371/journal.pone.0291689)
Supplement: S1 Text — (PDF) [file pone.0291689.s012.pdf]

S1 Text. Elaboration of data pre-processing

In this appendix, we detail all the data pre-processing that was carried out as a necessary prerequisite for modelling. First, we performed some data transformation and feature engineering to obtain the desired features for our analysis and modelling, namely:

- Based on the course catalogue data, we created a variable for whether a course had a group examination element, as well as dummy variables for potential changes in examination format (due to the closure).
- From the HR data, we grouped several minor departments into an "other" department.
- Based on the grade data, we created historical grade point averages for each student and each course.
- Based on student background data, we created a student age variable based on the student's date of birth, and we created a student nationality variable taking the values "Danish" or "International".
- Based on the data for student evaluation of teachers, we created variables for historical evaluation of individual teachers and individual courses.
- Based on the survey data we created a dummy variable for whether teachers have children (under the age of 15). Moreover, we replaced NAs with 0 for some of the survey answers in the case NAs corresponded to none. We also created a dummy variable for whether a teacher engaged in peer consulting (this included making a few manual encoding of teachers answering "other"). For the Likert scale questions: "Q3_1", "Q3_2", "Q7", "Q8", "Q9", "Q13", "Q15", "Q17", "Q25", "Q27", "Q29", "Q30", "Q31", "Q32", and "Q37", we replaced the answer 0 ("Do not know") with the mean answer for the given question. Finally, we created dummy variables for teachers receiving support from the IT department, their own department, their study board, and the Teaching and Learning unit at the university.

In addition to data transformation and feature engineering, we also had to filter out part of the data either as a prerequisite for the above feature engineering or because part of the data was deemed irrelevant for our analysis. More specifically, we filtered the data to only include data points for which:

- the exam type was "ordinary"
- the grade was numeric (contrary to only "pass"/"non-pass")
- the student had a historical GPA
- the course had historical grades
- the student got a passing numeric grade
- the student's age was within 3 standard deviations from the mean age (This only removed older students and removed less than 2% of the students)
- the course was not a master or bachelor thesis (Thus, we only included regular courses)
- the teacher answered the teacher survey

- the course needed to change due to the lockdown, according to the teacher (This removed courses that finished before the lockdown happened, for instance).

In addition to the above feature engineering and filtering, additional filtering was performed for each of the two analyses, students' grade performance and SET-score.

For the grade model, we removed all data points with missing values that were still in the dataset. This left us with 15,279 data points concerning 6,702 students, 162 teachers, and 144 courses. All variables were made into numerical variables, and categorical variables were made into dummy variables.

For the SET-score model, we removed all data points with missing values that were still in the dataset. As this data consists of students' evaluation of their teachers, we have substantially less data, since only some students filled out the teacher evaluations. Thus, we are left with 2336 data points about 1581 students, 114 teachers, and 114 courses. As in the case for the other model type, all variables were made into numerical variables, and categorical variables were made into dummy variables.
