# Supplementary material for: Student and teacher performance during COVID-19 lockdown: An investigation of associated features and complex interactions using multiple data sources
Source: PLoS One. 2023 Oct 25;18(10):e0291689. doi: 10.1371/journal.pone.0291689 (PMC10599549; doi:10.1371/journal.pone.0291689)
Supplement: S2 Text — (PDF) [file pone.0291689.s013.pdf]

S2 Text. Description of alternative model results

In this appendix, we elaborate on the alternative models compared to the main models.

For the models without variables based on the teacher survey, we observe a drop in R-square for the linear regression model (from 0.24 to 0.21). However, removing the variables based on the teacher survey only have a minor consequence for the effect of the remaining variables. Still, we do see that the effect of the number of courses taught and the age of the teacher become significantly positive, while Danish citizenship and the fact that the course is taught in the third semester becomes negatively significant. For the staff category, the effect of being a young VIP changes from slightly negative and insignificant to being positive and significant, while being a part time employee (DVIP) changes from insignificantly positive to significantly negative. For the random forest model, we observe that the r-square only decrease very slightly from 0.32 to 0.31. Furthermore, we see almost the same order of variable importance in the top, with the obvious difference that teacher survey variables do not appear in the top of the model without these variables. An interesting difference though, is that the number of courses variable jumps from number 15 to number 5 in the model with out teacher survey data. Since we have left out a large number of variables in these alternative models it is expected that the remaining variable will become more important. However, it is interesting that there is a seemingly effect of the number of courses a teacher have to teach that may work for a proxy of other variables from the teacher survey. Which variables is left for future research.

Based on data about which teachers a student is assigned to evaluate, we assigned a weight to each student-course-teacher data point, to represent the fact that a course can have multiple teachers. [1] With this weighting of the data, the linear regression model slightly increase its r-square (from 0.242 to 0.254), while the random forest model actually drops in r-square (from 0.289 to 0.287). The changes in effect for both the linear regression and random forest model are minor. In the linear regression model a few variables on the borderline of significance changes: *Will use no tools in future, teacher* becomes insignificant, *Female, teacher* becomes positively significant, while *Course taught in Q4, teacher* becomes negatively significant.

For the first alternative model for SET, which exclude the overall course evaluation feature, we see a significant drop in performance as r-square drops from 0.52 to 0.23 in the linear regression model and from 0.48 to 0.18 in the random forest model. With regards to the effects, we see that the historical SET-score of the teacher increases in the linear model, while dropping a bit in importance in the random forest model. For the linear regression model, *Assessment of students in course, teacher* suddenly become insignificant, while *Time usage on course, teacher* and *Assistant professor, teacher* become positively significant. For the random forest model the age of students become substantially more significant and moves to be the most significant. Moreover, *Self-assessed efficiency, teacher* increases its importance relative a bit, while *Course kept teaching design, teacher* decreases its relative importance slightly.

Including a historical course mean evaluation variable in the linear regression model for SET-score has no effect on the performance (r-square) nor the effect of the variables. For the random forest model, see actually see a drop in r-square from 0.48 to 0.42, while the new variable becomes the fourth most important. Otherwise, there is only little change in relative importance in the top.

Excluding all features from the teacher survey result in a performance drop in R-square from 0.52 to 0.49 for the linear regression model and from 0.48 to 0.45 for the

[1] When a course have multiple teachers, multiple student-course teachers data points are created, thereby increasing the presence of that particular student and course. To avoid this skewing of data we created equal fractional weights such that the effect of a student in a course with one teacher has the same effect a student in a course with three teachers.

random forest model. Removing the mentioned variables has no interesting effect on the linear regression estimates among the significant ones. (A few of the departments control features becomes significant though.) For the random forest model, we see that variable importance increases for all variables (as there are much fewer variables), however, there are only minor changes in the order of importance among the insignificant features (*No of courses, teacher*, *Female, student*, *Danish citizen, teacher*, and *Danish citizen, student* move up in relative importance).

Finally, we train a random forest classification model where we treated the target SET-score as a categorical variable. We naturally cannot calculate a R-square for this model, but on the training data, we receive an accuracy of 0.756, a precision of 0.772, a recall of 0.564, and a f1-score of 0.623. When we look at variable importance we see an overall drop in importance, however, a few relative changes also occur. *Age, student* becomes more important and move up one place to the second place, while *Historical SET-score, teacher* becomes less important and move down one place to a third place. Moreover *Female, student* and *Danish citizen, student* become substantially more important and moves up to a fourth and a sixth place.
