# Supplementary material for: Student and teacher performance during COVID-19 lockdown: An investigation of associated features and complex interactions using multiple data sources
Source: PLoS One. 2023 Oct 25;18(10):e0291689. doi: 10.1371/journal.pone.0291689 (PMC10599549; doi:10.1371/journal.pone.0291689)
Supplement: Attachment — Submitted filename: Response to Reviewers.docx [file pone.0291689.s014.docx]

# Response to Reviewers

Manuscript: PONE-D-23-17797

Title: Student and teacher performance during COVID-19 lockdown: An investigation of associated features and complex interactions using multiple data sources

| **Reviewer comment** | **Authors’ response** |
| --- | --- |
| 1. Please ensure that your manuscript meets PLOS ONE's style requirements, including those for file naming. | We have gone through the PLOS ONE's style requirements carefully one more time. We see that the way we named our figure and Supporting information files where wrong. We have corrected this to the best of our knowledge. We have also changed how we structure the Supporting information at the end of the paper. We hope that we now live up to all PLOS ONE’s style requirements |
| 2. Please include your full ethics statement in the ‘Methods’ section of your manuscript file. In your statement, please include the full name of the IRB or ethics committee who approved or waived your study, as well as whether or not you obtained informed written or verbal consent. If consent was waived for your study, please include this information in your statement as well. | We have updated our ethic statement, now in section 2.1. Here we have described that consent was obtained for the survey data and explained how the study was reported to the Danish Data Protection Agency and approved. |
| 3. Please include a copy of Table 6 which you refer to in your text on page 18. | We realized that our reference at page 18 to Table 6 should have been a reference to Table 3, which is included in our paper. We have now made this correction. |
| 4. Please include captions for your Supporting Information files at the end of your manuscript, and update any in-text citations to match accordingly. | We have updated the captions for our Supporting Information files at the end of the paper as well as all the in-text citations in our paper to the Supporting Information. Hopefully, we now live up to the formatting. |
| 5. Please review your reference list to ensure that it is complete and correct. If you have cited papers that have been retracted, please include the rationale for doing so in the manuscript text, or remove these references and replace them with relevant current references. Any changes to the reference list should be mentioned in the rebuttal letter that accompanies your revised manuscript. If you need to cite a retracted article, indicate the article’s retracted status in the References list and also include a citation and full reference for the retraction notice. | We have gone carefully through the reference updating information about all papers that has since been published etc. We found no papers that were retraced, but we found working papers that are now published. We updated the information about those. |
| While revising your submission, please upload your figure files to the Preflight Analysis and Conversion Engine (PACE) digital diagnostic tool, https://pacev2.apexcovantage.com/. PACE helps ensure that figures meet PLOS requirements. To use PACE, you must first register as a user. Registration is free. Then, login and navigate to the UPLOAD tab, where you will find detailed instructions on how to use the tool. If you encounter any issues or have any questions when using PACE, please email PLOS at figures@plos.org. Please note that Supporting Information files do not need this step. | We have uploaded all our figures to the PACE tool and renamed them in accordance with the PLOS ONE requirements. |
| ***Reviewer 1*** |  |
| You have a very good work, deserving publication. Nonetheless, some amendments in the format are due to admit publication. | Thank you very much and thank you for you your constructive comments that helped improve our paper. |
| The main problem is the epistemological structure (why the article was conceived and how the study was developed). I suggest the following structure of objectives: (i) research gap; (ii) research question; (iii) purpose of the article; (iv) intermediate objectives; (v) assumptions or hypo; and (vi) research method. This structure must appear in the introduction (you have some parts, but it needs to be put together).  The research gap must be created by a systematic literature review that provides 'holes' in the state of knowledge on the topic. I believe that a full review should not be done, but an analysis of about 5-8 studies on the topic under discussion. You can find some examples, which will show the relevance of the issue, as it is indeed a topic of current, relevant research. At the end of the justification you should write something like: According to what we were able to find, there are no studies referring and reporting on ... With this you have therefore proven that the issue is relevant, and you have also proven that your study does indeed fill a research gap. | We have rewritten our introduction entirely in the format suggested by the reviewer. Moreover, we have added a literature review in a separate section just after the introduction.  We are deeply grateful for the reviewer’s suggestion to revise our introduction. We believe it have made our purpose much more clear and helped improve our paper significantly.  In an attempt to keep the paper of a reasonable length, we have made the literature review relatively short, but we hope it fulfills the reviewers request and show the research gap we attempt to bridge. Otherwise, we are of course willing to extend it on your instructions. |
| I propose the classic division of a scientific article:  Introduction  Literature review  Research methodology  Results  Discussion  Conclusions  Your division into many chapters and subchapters creates chaos in your work. | We have now changed the paper such that there are six main sections:  1 Introduction  2 Literature review  3 Materials and methods  4 Results  5 Discussion  6 Conclusion  We have also removed some of the subsection headings. However, we have left some subsection headings as we find that they help make the themes clear, for instance in the discussion. However, if Reviewer 1 finds that there is still too may subsections, we can of course cut down even further. |
| ***Reviewer 2*** |  |
| Dear Author  Paper is good (very good - my opinion)  The research topic is a summary of the situation after COVID-19. The research is implemented correctly. | Thank you very much for your review and positive outlook at our paper and research. |
| As a reviewer I am missing a completed literature review after the Introduction | We have now added a Literature review in a separate section after the introduction. In an attempt to keep the paper of a reasonable length, we have made it relatively short, but we hope it fulfills your request. Otherwise, we are of course willing to extend it on your instructions. |
| One note: Figures have a poor resolution | Thank you for noticing. After uploading all our figures to the PACE tool as described above, the resolution should be much better in all our figures. |
